# Analysing time-use composition as dependent variables in physical activity and sedentary behaviour research: different compositional data analysis approaches

**DOI:** 10.1186/s44167-023-00033-5

**Published:** 2023-11-02

**Authors:** Philip von Rosen

**Affiliations:** Department of Neurobiology, Care Sciences, and Society (NVS) Division of Physiotherapy, Karolinska Institutet, Alfred Nobels Allé 23, Huddinge, SE-141 83 Sweden

**Keywords:** Compositional data analysis, Ilr coordinates, Isometric log-ratio, Outcome

## Abstract

Recently, there has been a paradigm shift from considering physical activity and sedentary behaviour as “independent” risk factors of health to acknowledging their co-dependency and compositional nature. The focus is now on how these behaviours relate to each other rather than viewing them in isolation. Compositional data analysis (CoDA) is a methodology that has been developed specifically for compositional data and the number of publications using CoDA in physical activity and sedentary behaviour research has increased rapidly in the past years. Yet, only a small proportion of the published studies in physical activity and sedentary behaviour research have investigated the time-use composition as dependent variables. This could be related to challenges regarding the interpretation of the results and the lack of guidelines for deciding which statistical approach to use. Therefore, in this paper, four different approaches for analysing the time-use composition as dependent variables are presented and discussed. This paper advocates that the aim of research should guide how the dependent variable is defined and which data analysis approach is selected, and it encourages researchers to consider analysing time-use components as dependent variables in physical activity and sedentary behaviour research.

**Supplementary Information:**

The online version contains supplementary material available at 10.1186/s44167-023-00033-5.

## Background

Traditionally, epidemiologists have explored time spent in different movement behaviours across a day, such as time spent in sleep, sedentary behaviour, light physical activity and moderate-to-vigorous physical activity, in relation to health outcomes. Recently, there has been a paradigm shift from considering movement behaviours as “independent” risk factors of health to acknowledging their co-dependency and compositional nature [1–3]. Instead of exploring the health effects of these behaviours in isolation or with partial adjustments for one another, the importance of adequately adjusting for all parts of the time-use composition has been highlighted [2, 3]. In this perspective, time spent in movement behaviours can be seen as a composition, a vector of parts. A composition is a series of positive values (e.g. amounts of time spent in different movement behaviours), that adds up to the total available time in a given period (e.g. day, week, month) [4].

Given that the amounts of time spent in movement behaviours are parts of a finite total, these variables are perfectly multicollinear [4]. Therefore, a change in time spent in one behaviour displaces time spent in, at least, one of the remaining behaviours in the composition. The compositional parts are commonly expressed as proportions (i.e. relative to the total time). This means they are bounded from zero to one (or 1 to 100 for percentages) and exist in a non-Euclidean space [4]. Compositional data analysis (CoDA) is a methodology that has been developed specifically for compositional data, and the number of publications using CoDA in physical activity and sedentary behaviour research has increased rapidly in the past several years. Analysing time-use compositions using statistical techniques that have been designed for non-compositional data may lead to misleading inferences [5, 6]. Therefore, several methodological papers have recommended to use CoDA when analysing time-use compositions in physical activity and sedentary behaviour research [2, 3, 6].

A widely used CoDA approach starts with a log-ratio transformation of compositional parts. While there are several types of log-ratios available, isometric log-ratios (*ilr*) [7] have been most used in physical activity and sedentary behaviour research. Applying the *ilr* transformation on a composition results in one less *ilr* coordinate compared to the number of compositional parts (e.g. two *ilr* coordinates will be calculated from a 3-part composition) [7]. The first *ilr* coordinate (*ilr*_*1*_) represents time spent in one behaviour relative to the remaining behaviours. After this transformation, standard statistical techniques can be applied to the *ilr* coordinates [8].

In recent years, CoDA has drawn significant attention from researchers in the field of physical activity and sedentary behaviour research. A simple search through titles and abstracts of articles indexed in PubMed using the following syntax “compositional data analysis” AND “physical activity” shows a rapid increase in the number of publications in recent years. The first study in this field was published in 2015, followed by zero, four, 16 and 22 studies the following years to 2019. In 2020–2022, 37 to 41 studies have been published yearly. For example, CoDA has been applied to explore the association between movement behaviours and all cause-mortality, non-communicable diseases, biomarkers and movement skills [9–12]. However, surprisingly few studies have applied CoDA when analysing movement behaviours as dependent variables. Of the 151 studies published in 2015–2022, based on the above-mentioned literature search, only around 12% (n = 18) analysed movement behaviours as the dependent variables [13–30]. This is a surprisingly small proportion of the publications, given that a common aim in epidemiological research is to explore changes in movement behaviours across time, differences in movement behaviours between groups or determinants for movement behaviours [31]. It has been much more common to apply CoDA for analysing movement behaviours as independent variables in regression models. By entering all *ilr* coordinates as independent variables in a regression model, the model attempt to account for all portions of time spent in each behaviour that add up to a finite time. Thereby accounting for their combined effect on the dependent variable. However, analysing the composition of movement behaviours as the dependent variables may not be as straightforward and several different approaches could be considered. In this paper, we first describe how a time-use composition can be defined. Secondly, four different statistical approaches are presented and discussed based on regression models; (i) the first pivot coordinate (*ilr*_*1*_), (ii) the first *pivot* coordinate adjusted for the remaining *pivot* coordinate(s), (iii) all *ilr* coordinates separately analysed, (iv) all *ilr* coordinates “stacked” into a single variable.

### Defining a time-use composition

**Fig. 1:**
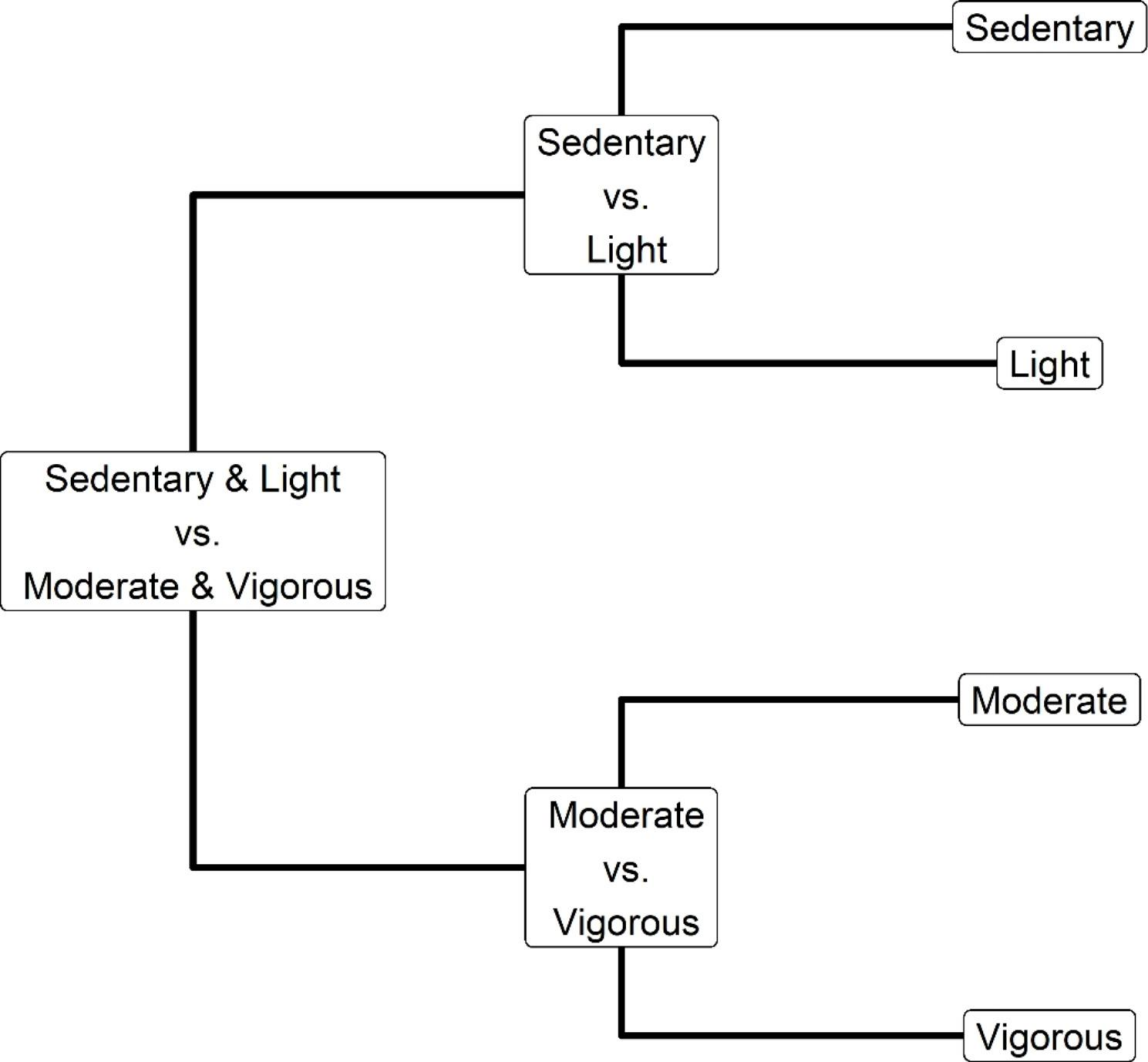
Example of sequential binary partition of four movement behaviours, representing time spent sedentary behaviour, light, moderate and vigorous physical activity, contrasted in three coordinates. Note that the partitions results in non-overlapping groups

Typically, the time-use composition is defined as pivot coordinates or balance coordinates in physical activity and sedentary behaviour research. Using pivot coordinates, the coordinates are defined as one movement behaviour relative to the remaining behaviours. This approach has been used and described in several studies [3, 4, 10, 11]. Balances coordinates are a more flexible way of defining the time-use composition, focusing on the relationship between different parts of the movement behaviours. Sequential binary partition (SBP) can be used to partition movement behaviours into groups, leading to interpretable coordinates of the compositional data [32]. SBP defines an orthonormal basis that splits the composition into a series of non-overlapping groups (in our case groups of movement behaviours). This process is then repeated until each of the movement behaviours forms a single-part “group” (Table 1). Consequently, an SBP consists of groups with different combinations of movement behaviours and the coordinates can be seen as contrasts that are interpretable as one or several movement behaviours relative to one or several other behaviours. After the groups of parts are defined, which can be visualized using a balance dendrogram (Fig. 1), the *ilr* transformation can be applied. The new *ilr* coordinates (balances) can then be used as dependent variables. For example, a composition of time spent in sedentary behaviour, light, moderate and vigorous physical activity can be split into two groups: one including sedentary behaviour and light physical activity and the other including moderate and vigorous physical activity (*ilr*_*1*_). The first group can then be split into time spent in sedentary behaviour relative to light physical activity (*ilr*_*2*_) and the second group can be split into time spent in moderate physical activity relative to time spent in vigorous physical activity (*ilr*_*3*_). Expert opinion or a data-driven approach to inform meaningful balances. Each of the balances can then be entered as a dependent variable in a regression model, with or without adjustments for the remaining balances. Balances has been used to explore time-use composition among pre-schoolers [12], changes in time-use composition during the transition to retirement [30] and followed a physical activity intervention [26]. The “Balance” package in R can be used for this purpose [33].

**Table 1:** Example of sequential binary partition for four movement behaviours. The value “1” stands for inclusion in the nominator of a coordinate, the value “-1” stands for inclusion in the dominator of a coordinate, and the value “0” indicates that the part is not included to the coordinate

Coordinate	Coordinate 1 Sedentary behaviour & Light vs. Moderate & Vigorous	Coordinate 2 Sedentary behaviour vs. Light	Coordinate 3 Moderate vs. Vigorous
**Sedentary behaviour**	1	1	0
**Light**	1	-1	0
**Moderate**	-1	0	1
**Vigorous**	-1	0	-1

### Approach I: the dependent variable is the first pivot coordinate (*Ilr*_*1*_)

Of the transformed coordinates, *ilr*_*1*_ is the simplest and most interpretable variable to use as the dependent variable. *Ilr*_*1*_ represents time in one behaviour relative to times in all the remaining behaviours. Since, the remaining *ilr* coordinates (i.e. *ilr*_2,_*ilr*_3…_*ilr*_D−1_) do not contain the first part of the composition, we can interpret *ilr*_*1*_ as the relative importance of one behaviour with respect to a (geometric) average of the remaining behaviours [3]. Consequently, if *ilr*_*1*_ is used as the dependent variable, the model will simply explain which independent variables are associated with the dominance of one behaviour over the remaining behaviours. However, interpreting predicted values for *ilr*_*1*_ may be difficult. By applying the inverse *ilr* transformation_1_, time in a specific movement behaviour can be obtained. Note that perturbations of compositional parts can be performed before the log-ratio transformation, so that the numerator in *ilr*_*1*_ is time spent in any of the movement behaviours. For instance, if you are analysing a 3-part composition, you can create three pairs of *ilr* coordinates and analyse each *ilr*_*1*_ separately. This approach has been used to evaluate the effect of a randomized trial on time-use composition among office workers [21], people with diabetes [34] and in a cross-sectional study explore the association between occupational and leisure time physical activity among workers [35].

### Approach II: the dependent variable is the first *pivot* coordinate adjusted for the remaining *pivot* coordinate(s)

Before conducting a regression analysis, *ilr*_1_ can be statistically adjusted for the remaining *ilr* coordinates, that is, the “influence” of the remaining *ilr* coordinates (i.e. *ilr*_2,_*ilr*_3_, _…_, ilr_D−1_) can be statistically removed from *ilr*_1_. Thereby, it is possible to partially adjust or fully adjust *ilr*_*1*_. By regressing *ilr*_*1*_ on *ilr*_*2*_ (or other *ilr* coordinates), the unexplained variance of *ilr*_*1*_ will be left, unexplained from *ilr*_*2*_. The residuals represent the variance of *ilr*_*1*_ that is unexplained of the remaining *ilr* coordinates, and they can then be used as the dependent variable. This changes the interpretation of the model and may change the results of the analysis drastically, especially if *ilr*_*2*_ is associated with *ilr*_*1*_ in the final model. For instance, if age explains 60% of the variance of *ilr*_*1*_, adjusting *ilr*_*1*_ for *ilr*_*2*_ will likely reduce the explained variance if age is highly related to *ilr*_*2*_. If *ilr*_*2*_ is not related to age, adjusting *ilr*_1_ for *ilr*_2_ might increase the percentage of explained variance. Instead of adjusting *ilr*_1_ for the remaining *ilr* coordinates, it is also possible to include the *ilr* coordinates as independent variables in the regression model to account for their influence. Important to note is that it is likely more difficult to back-transform the data after statistical adjustment of one or several *ilr* coordinates. However, this approach might be useful when the study aims to explore in detail how independent variables are associated with a dependent variable, account for time spent in other movement behaviours.

### Approach III: the dependent variables are separately analysed for all *ilr* coordinates

The *ilr* coordinates can be analysed in separate regression models, e.g. a regression model is performed for each *ilr*. By predicting values in each model, results in that predicted values for all *ilr* coordinates are estimated. The inverse *ilr* transformation can then be applied, resulting in that time in each movement behaviour being estimated. This approach has been used when exploring changes in time-use composition in a Thai population [22].

### Approach IV: the dependent variables are all *ilr* coordinates “stacked” into a single variablee

It is also possible to use all ilr coordinates as the dependent variable in a single regression model. This can be done by restructuring the dataset from a wide format to a long format, leading to all values for the *ilr* coordinates being placed in a single column. An indicator variable is then used to define which data belongs to which *ilr* coordinate. Therefore, there will be as many levels of the indicator variable as there are *ilr* coordinates. The indicator is then used as an independent variable in the model. By including interaction terms between the indicator variable and each of the independent variables in the model, the association with each *ilr* coordinate can be explored separately. However, as we have repeated measures for each participant (one value for each *ilr*) we need to use a model that can handle correlated observations, such as a mixed model that can contain both fixed and random effects. The advantage of this approach is that all *ilr* coordinates can be explored simultaneously in one model. As the dependent variable is based on several *ilr* coordinates, separate random effects for each *ilr* are possible to specify, which may increase the complexity of the model, while the accuracy of the model may improve. This approach has previously been used in a secondary analysis of a randomised trial, aiming to explore changes in medication load on 24-hour activity composition [36].

## Methods

The publicly available NHANES 2003–2004 dataset was used to present examples of the four abovementioned approaches. Details on data collection in NHANES 2003–2004 have been described previously [37]. Participants were instructed to wear an ActiGraph 7164 accelerometer (ActiGraph, Shalimar, FL) on the right hip for seven consecutive days, to capture time in different intensities of physical activity. The device was set to sampling counts per 1-minute epochs and the *nhanesaccel* package for R *(*release 4.1.3; R Core Team, 2015, Vienna, Austria*)* was used to process the accelerometer data. In this analysis, data on time spent in sedentary behaviour (0–99 counts), light (100–759 counts), moderate (760–2019 counts) and vigorous physical activity (> 2000 counts) were used as dependent variables and age of the participants was used as the independent variable. The age variable was centered by subtracting the mean (average) value, to facilitate interpretation of the coefficient. A valid day was defined as 10 or more hours of wearing an accelerometer and participants with records of 4 or more valid days were included in the data analysis. This resulted in a sample of 6340 participants.

Time spent in sedentary behaviour, light, moderate and vigorous physical activity was transformed into *ilr* coordinates. Given that a 4-part composition was used, each movement behaviour was then represented by three *ilr* coordinates (z1, z2 and z3). Four different types of models were fitted where the independent variable was age and the dependent variable was (i) the first pivot coordinate (*ilr*_*1*_), (ii) the first *pivot* coordinate adjusted for the remaining *pivot* coordinate(s), (iii) all *ilr* coordinates separately analysed, (iv) all *ilr* coordinates “stacked” into a single variable. To provide an example of balance coordinates, coordinates were defined as sedentary behaviour and light physical activity relative to moderate and vigorous physical activity (*ilr*_*1*_), sedentary behaviour relative to light physical activity (*ilr*_*2*_) and moderate physical activity relative to vigorous physical activity (*ilr*_*3*_). For comparison purposes, a model with absolute time as the dependent variable was also fitted. In all models, linear regression was used, except for approach “iv”, where a linear mixed model was applied. The *ilr*_*1*_ was back-transformed to the unbounded space by applying the inverse *ilr* transformation. As values for all *ilr* coordinates are needed in the *ilr* transformation, age-adjusted arithmetic mean values of *ilr*_*2*_ and *ilr*_*3*_ were calculated. In the model with absolute time as the dependent variable, the predicted values for all behaviours were transformed into percentages. R code for all analyses is provided as a Supplementary File.

## Results

In Table 2, the results for all models are shown. Age was significantly associated with absolute time and relative time in all movement behaviours in all models. Even if the point estimates differed across models, the overall results were similar, i.e. higher age was associated with lower time spent in light, moderate and vigorous physical activity, and higher time spent in sedentary behaviour. However, in approach III & IV higher age was associated with lower time spent in sedentary behaviour, and higher time spent in light physical activity.

**Table 2:** Regression models with age, centered, as independent variable and different combinations of time spent in sedentary behaviour, light, moderate and vigorous physical activity, as the dependent variable

Regression models				
**Four linear regression models with absolute time as dependent variable**				
**Dependent variable**	**β**	**SE**	**P-value**	**R**^**2**^
Sedentary behaviour	2.30	0.08	< 0.001*	0.11
Light	-1.09	0.06	< 0.001*	0.05
Moderate	-0.62	0.01	< 0.001*	0.24
Vigorous	-0.05	0.002	< 0.001*	0.08
**Approach I: Four linear regression models with relative time (*ilr1*) as dependent variable**				
**Dependent variable**	**β**	**SE**	**P-value**	**R**^**2**^
Sedentary behaviour_*ilr*^*1*^	0.04	0.001	< 0.001*	0.38
Light_*ilr*_*1*_	0.03	0.001	< 0.001*	0.31
Moderate_*ilr*_*1*_	-0.01	0.0003	< 0.001*	0.13
Vigorous_*ilr*_*1*_	-0.05	0.001	< 0.001*	0.35
**Approach II: Four linear regression models with relative time (unexplained variance of *ilr1*) as dependent variable**				
**Dependent variable**	**β**	**SE**	**P-value**	**R**^**2**^
Sedentary behaviour_*ilr*_*1*_^a^	0.01	0.0005	< 0.001*	0.09
Light_*ilr*_*1*_^a^	0.005	0.0003	< 0.001*	0.04
Moderate_*ilr*_*1*_^b^	-0.003	0.0003	< 0.001*	0.02
Vigorous_*ilr*_*1*_^b^	-0.009	0.0006	< 0.001*	0.003
**Approach III: Three linear regression models with all *ilr* coordinates analysed separately**				
**Dependent variable**	**β**	**SE**	**P-value**	**R**^**2**^
Sedentary behaviour_*ilr*_*1*_	0.04	0.0006001	< 0.001*	0.38
Sedentary behaviour_*ilr*_*2*_	0.04	0.0007001	< 0.001*	0.35
Sedentary behaviour_*ilr*_*3*_	0.03	0.0006001	< 0.001*	0.26
**Approach IV: One linear mixed model with values of *ilr1,ilr***_***2 ***_***and ilr***_***3 ***_**as dependent variable**				
**Independent variable**	**β**	**SE**	**P-value**	
Sedentary behaviour_*ilr*_*1*_	3.28	0.01	< 0.001*	
Light vs. moderate & vigorous_*ilr*_*2*_	4.19	0.01	< 0.001*	
Moderate vs. vigorous_*ilr*_*3*_	3.11	0.01	< 0.001*	
Age	0.04	0.01	< 0.001*	
Interaction: Age* Light vs. moderate & vigorous_*ilr*_*2*_	0.01	0.0004	< 0.001*	
Interaction: Age* Moderate vs. vigorous_*ilr*_*3*_	-0.01	0.0004	< 0.001*	
**Balance coordinates: Three linear regression models with *ilr *coordinates as dependent variable (created from sequency binary partition)**				
**Dependent variable**	**β**	**SE**	**P-value**	**R**^**2**^
Sedentary behaviour & Light vs. Moderate & Vigorous_*ilr1*	0.03	0.0005	< 0.001*	0.35
Sedentary vs. Light_*ilr*_*2*_	0.01	0.0002	< 0.001*	0.11
Moderate vs. Vigorous_*ilr*_*3*_	0.03	0.0006	< 0.001*	0.26
^a^ Unexplained variance for time spent in moderate vs. vigorous physical activity ^b^ Unexplained variance for time spent in light vs. vigorous physical activity *ilr1*, first isometric log-ratio coordinate; ilr_2_, second isometric log-ratio coordinate; *ilr*_*3*_, third isometric log-ratio coordinate; SE, standard error; β, slope, R², coefficient of determination.				

**Fig. 2:**
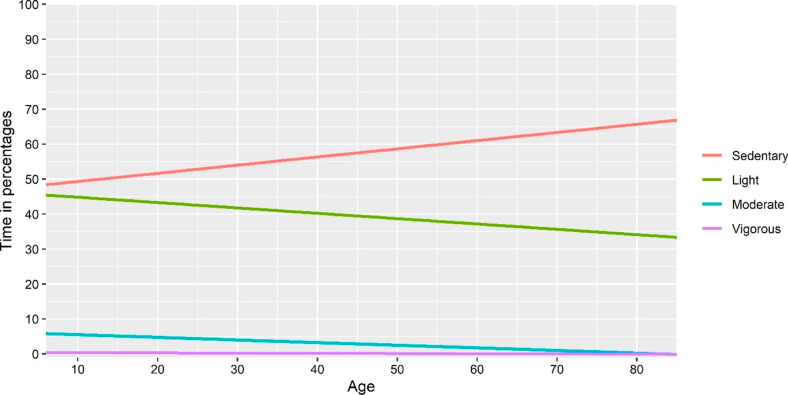
Association between age and relative time in movement behaviours based on a linear regression model with absolute time in different behaviours as the dependent variable

**Fig. 3:**
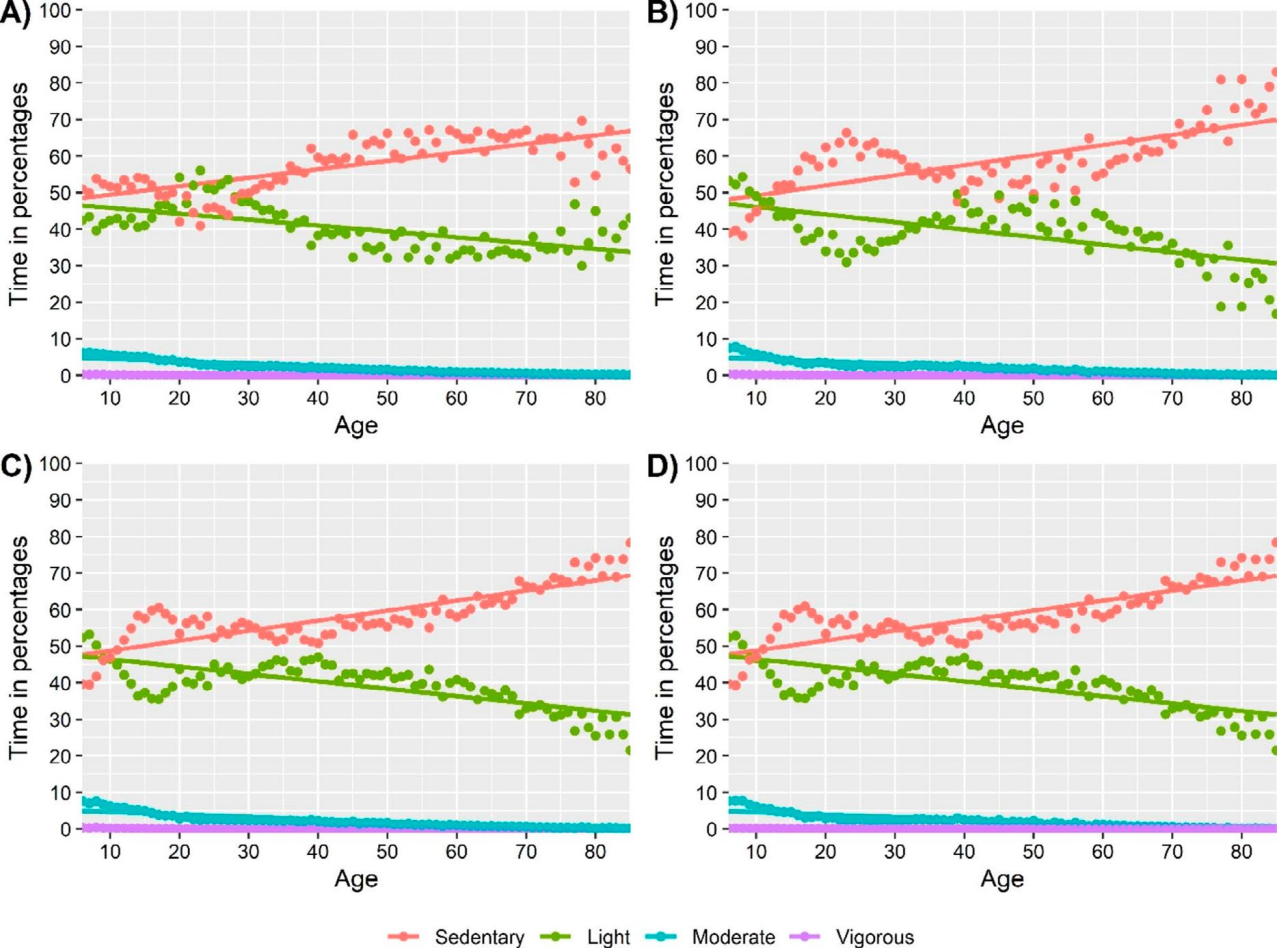
Association between age and relative time in movement behaviours based on a linear regression model with the isometric log-ratio_1_ coordinate for **A**) Sedentary behaviour, **B**) Light, **C**) Moderate, **D**) Vigorous physical activity as the dependent variable

**Fig. 4:**
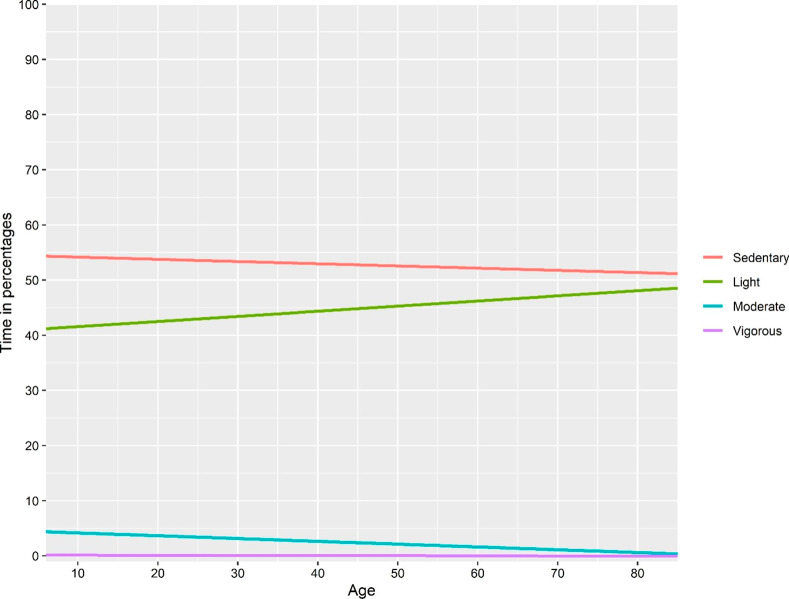
Association between age and relative time in movement behaviours, where the isometric log-ratio_1 − 3_ were separately analysed as the dependent variable in linear regression models

**Fig. 5:**
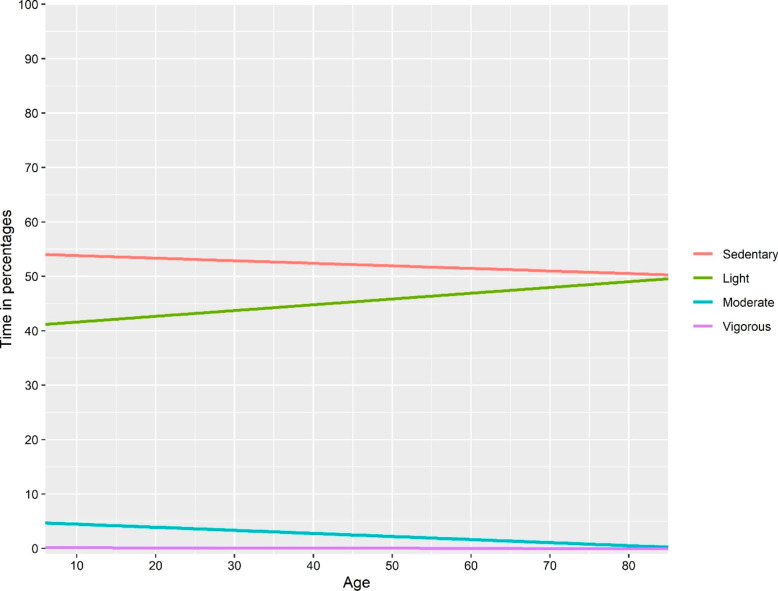
Association between age and relative time in movement behaviours based on a linear mixed regression model, including the values of isometric log-ratio_1 − 3_ coordinate as the dependent variable

**Fig. 6:**
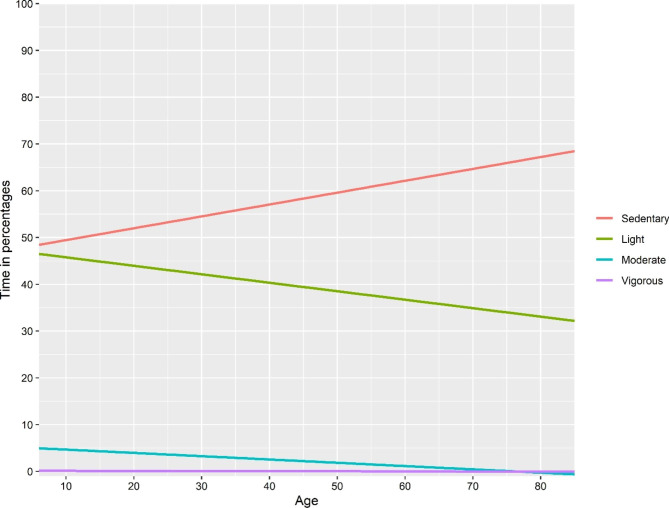
Association between age and relative time in movement behaviours based on three linear regression models, where the dependent variables are balances. The balance coordinates were sedentary behaviour and light physical activity relative to moderate and vigorous physical activity (isometric log-ratio_1_), sedentary behaviour relative to light physical activity (isometric log-ratio_2_) and moderate physical activity relative to vigorous physical activity (isometric log-ratio_3_)

The explained variance seemed to be higher in approach I & III compared with the model with absolute time as the dependent variable, for all dependent variables except moderate physical activity. The explained variance seemed to be lower in approach II, compared to approach I. Line plots based on fitted regression models of “absolute time”, approach I and approach III-V are presented in Figs. 2, 3, 4, 5 and 6. Note that the point estimates differed in Fig. 3A-D for different combinations of *ilr*_1 − 3_, even if the trend of the regression lines was similar.

## Discussion

Even if the overall results for the four different approaches were similar, our results illustrate that the results may vary depending on how the time-use composition is defined and treated in the analysis. In our case, the model with absolute time as the dependent variable showed similar results as approach I-IV, demonstrating that age was associated with the absolute and relative time of different movement behaviours. Based on the coefficient of determination, approach I and III seem to explain the highest proportion of variance. However, the current study cannot state which model provides the most realistic estimates. Since the time-use composition is defined and treated differently across the approaches, direct comparisons are not possible. However, several considerations can be suggested and a summary of the approaches with comments is presented in Table 3.

**Table 3:** Four approaches to include time-use composition as the dependent variable in regression models with comments

Approach	Pros	Cons
Approach I: *Ilr*_*1*_ is the dependent variable	Easy to interpret based on predicted values, information criteria and parameter estimates.	Ignore the remaining *ilr* coordinates.
Approach II: The dependent variables is the unexplained variance (from other *ilr* coordinates) of *ilr*_*1*_	Possible to statistically adjust for one or several *ilr* coordinates. Information criteria and parameter estimates can be used to assess the model.	Difficult to interpret predicted values.
Approach III: All *ilr* coordinates separately analysed as the dependent variable	Results can be interpret based on predicted values, information criteria and parameter estimates.	Use all *ilr* coordinates, but in separate models.
Approach IV: All *ilr* coordinates “stacked” as the dependent variable	Results can be interpret based on predicted values, information criteria and parameter estimates.	Extra parameters are needed to be estimated to distinguish between *ilr* coordinates.
*Ilr*, isometric log-ratio.		

How to define and treat the time-use composition should be based on the aim of the study. If a study aims at exploring the determinants of a specific movement behaviour relative to the remaining behaviours, approach I and II can be used. If the aim concerns determinants or between-group comparisons of time-use composition, approach III and IV can be used as estimates for all movement behaviours can be derived. Further, if a study explores determinants for a composition of multiple movement behaviours, the use of balance coordinates offers a systematic process to define the composition of movement behaviour. Obtaining predictive values can be used to interpret the models in all approaches, although it might be more difficult for approach II. Information criteria (e.g. Akaike’s information criterion, Bayesian information criterion, coefficient of determination) and parameter estimates can be used to guide which model to choose and how to interpret the model. Besides, the approaches described in this paper, several studies have used multivariate analysis of variance (MANOVA) to explore differences in time-use composition between groups [5, 13]. Regardless of which approach is chosen, the model assumptions still need to be checked despite how the time-use composition is defined and treated.

## Conclusions

This paper advocates that the aim of research should guide how the dependent variable is defined and which data analysis approach is selected, and it encourages researchers to consider analysing time-use components as dependent variables in physical activity and sedentary behaviour research. Examples of different approaches for using the composition of time-use as dependent variables have been exemplified, discussed and codes for analyses have been provided. This is believed to strengthen the physical activity and sedentary behaviour research field and increase our understanding of movement behaviours co-dependency in various research settings.

## Electronic supplementary material

Below is the link to the electronic supplementary material.

Supplementary Material 1

## Data Availability

Data are available in a public, open-access repository and can be accessed at the https://www.cdc.gov/nchs/nhanes/index.htm. R codes are available as a supplementary file.
